# Evaluation of the glycemic indices of three commonly eaten mixed meals in Okada, Edo State

**DOI:** 10.1002/fsn3.550

**Published:** 2017-11-24

**Authors:** Kingsley Omage, Sylvia O. Omage

**Affiliations:** ^1^ Department of Biochemistry College of Basic Medical Sciences Igbinedion University Okada Edo State Nigeria; ^2^ Department of Biochemistry Faculty of Life Sciences University of Benin Edo State Nigeria

**Keywords:** beans, glycemic index, glycemic load, mixed meal, plantain, rice

## Abstract

People do not generally eat single or individual meals; rather they eat mixed meals, consisting of two or more individual meals. These mixed meals usually have glycemic indices which differ from that of the individual food type. This study was aimed at evaluating the glycemic indices of three commonly consumed mixed meals eaten in Okada; rice and beans (test food 1), rice and plantain (test food 2), beans and plantain (test food 3). Two hundred and forty healthy subjects aged between 18 and 30 participated in this study. They were randomized into three groups of eighty persons each, and fed with the standard food (50 g glucose) on day one and one of the test foods on day two, after an overnight fast. Blood samples were taken at 0, 30, 60, 90, and 120 min after the food had been eaten. The results showed that the Glycemic Index (GI) values for the test foods were high: 86.60 (test food 1), 89.74 (test food 2), 86.93(test food 3). The incremental increase in blood glucose was monitored and calculated for each food and when compared with that of the standard food (glucose), there was significant differences (*p* < .036) for test food 1 and (*p* < .068) for test food 3; both at 30 min. At 120 min, no significant differences in blood glucose levels were observed (*p* > .05). The results from this study indicated that the GI of the mixed meals was affected by the constituent nutrient and the response is also affected by the proportion of each nutrient. Our findings show that the selected test foods (mixed meals) consumed in Okada have high GI values.

## 1 INTRODUCTION

In recent times, there has been an increased interest in the association of carbohydrate intake with human nutritional status, energy balance, and chronic disease risk (Cho et al., 2003; Jenkins et al., 2002; Liu et al., 2000; Oh, Willett, Fuchs, & Giovannucci, 2004). Carbohydrates are typically the primary energy source for most humans. However, there is controversy surrounding the optimal quantity and quality of carbohydrates that should be recommended for consumption (Schneeman, 2001). Developing appropriate dietary assessment methods for research studies that address these issues is challenging because carbohydrates differ in their ability to influence immediate and long‐term metabolic responses (i.e., postprandial glucose and insulin, and signaling molecules such as insulin‐like growth factors). These physiologic responses have important implications for energy balance, cardiovascular disease, and cancer (Jenkins et al., 2002; Michaud et al., 2002; Noakes, Keogh, Foster, & Clifton, 2005; Oh et al., 2004; Pi‐Sunyer, 2002). The glycemic load (GL) measure was introduced to capture information on the overall glycemic effect of the diet, which is believed to be the biologically relevant exposure in epidemiologic studies that examine associations of carbohydrate with disease risk (Brand‐Miller et al., 2003; Salmeron et al., 1997.).

Glycemic Index (GI) is a quantitative assessment of foods based on postprandial blood glucose response (Jenkin, Ocana, & Jenkins, 1992; Jenkins, Wolever, & Taylor, 1981), which is often expressed as a percentage of the response to an equivalent carbohydrate portion of a reference food (white bread or glucose) (Wolever et al., 2001). Carbohydrate foods consumed with same amounts of glucose produce different glycemic responses (Jenkins et al., 1981) depending on the nature of the food and type and extent of food processing. People do not generally eat single foods, but mixed meals or snacks made up of macronutrients. Several studies have been carried out to evaluate the effects of combinations of macronutrients on the GI. These studies have shown that; the higher the proportion of carbohydrate in a specific food, as opposed to protein and fat, the higher the GI and a mixed meal of carbohydrate, protein, and fat will have a different and variable glucose response depending on the proportions of each nutrient. Thus, the glucose responses of a food eaten alone or in combination with other foods differ considerably (Calle‐Pascual, Gomez, Leon, & Bordiu, 1988; Coulston, Hollenbeck, Swislocki, & Reaven, 1987; Laine, Thomas, Levitt, & Bantle, 1987). The insulin response to a carbohydrate food varies with the amount of fat, protein, or both, with which it were ingested.

Some of the common mixed meals eaten in Okada town, Edo State, are Rice and Beans, Rice and Plantain, Beans and Plantain. Rice, the seed of the grass species *Oryza sativa* (Asian rice) or *Oryza glaberrima* (African rice), is a cereal grain. It is the most widely consumed staple food for a large part of the world's population, especially in Asia. Rice is the most important grain in human nutrition and caloric intake, providing more than one‐fifth of the calories consumed worldwide by humans. Beans, *Phaseolus vulgaris* (navy/white beans), is a common name for the large plant seeds of several genera belonging to the family *Fabaceae* which is used for human or animal food. Beans are high in protein, complex carbohydrates, and iron. The balance of complex carbohydrates and protein provides a slow, steady source of glucose instead of the sudden surge that can occur after eating simple carbohydrates. *Musa paradisiaca* (Plantain) is a crop from the genus *Musa*. Its fruits are edible, and are generally used for cooking.

These common foods are often eaten in combinations so as to derive optimal nutrients from them. Although several studies have been carried out which examine the glycemic indices of the individual foods, there is dearth of information on studies evaluating the glycemic indices of combinations of these foods (mixed meals). Thus, the aim of this study was to evaluate the glycemic indices of three commonly consumed mixed meals in Okada Town, Edo State, Nigeria; rice and beans (test food 1), rice and plantain (test food 2), beans and plantain (test food 3).

## 2 MATERIALS AND METHODS

### 2.1 Test food

The three food combinations were selected as highly reproducible and acceptable by the subjects. The selected foods were bought and prepared in Okada Town, Edo State, Nigeria. The selected mixed meals were; rice and beans (test food 1), beans and plantain (test food 2), rice and plantain (test food 3).

### 2.2 Sample collection and processing

Rice grain (*Oryzae sativa),* bean seed (*Phaseolus vulgaris*), and ripe plantain (*Musa paradisiacal*) samples were all purchased from Okada market, Okada town, Edo State, Nigeria. The rice grain was washed with water and boiled for 40 min. The bean seed was selected from dirty particles and shafts, parboiled for 10 min and left to cook for 50 min using a pressure cooker. The ripe plantain was peeled from its skin, cut into smaller sizes and fried, using groundnut oil.

### 2.3 Proximate analysis

Proximate analysis was carried out for the three test foods. Total carbohydrate, ash, moisture content, crude fiber, crude fat and crude protein content were determined using the methods of the Association of Official Analytical Chemist (AOAC, 2003). This was done to determine the serving sizes of the test foods.

### 2.4 Determination of moisture content

#### 2.4.1 Principle

These methods rely on measuring the mass of water in a known mass of the sample.

#### 2.4.2 Procedure

The moisture content was determined by weighing the samples into moisture cans and placed in an oven at 80°C till constant weight of the sample was obtained (dried to a constant weight). They were brought out and allowed to cool in desiccators and reweighed (AOAC, 2003). Thus,

$$\text{Moisture}\;\left(\%\right)=\frac{\text{Weight of wet sample}\;-\;\;\text{weight of dried sample}}{\text{Weight of wet sample}}\times 100$$

### 2.5 Ash content determination

#### 2.5.1 Principle

Water and other volatile materials were vaporized and organic substances burned in the presence of oxygen in air to CO_2_, H_2_O, and N_2_.

#### 2.5.2 Procedure

Five grams each of the samples were transferred into a preweighed porcelain crucible and weighed. The crucible was then placed in a muffle furnace for 6 hr at 600°C to burn off all organic materials. The furnace was allowed to cool below 200°C and maintained at this temperature for 20 min. Then the crucible was placed in a desiccator with stopper top, allowed to cool and then reweighed to measure the ash content (AOAC, 2003).

$$\text{Ash}\;(\%)=\frac{\text{Weight of Ash}}{\text{Weight of sample}}\times 100$$

### 2.6 Crude protein determination

#### 2.6.1 Principle

The Kjeldahl method, which is the standard method for determining protein and other nitrogen containing compounds, was used. A food is digested with strong acid so that it releases nitrogen which can be determined by a suitable titration technique. The mount of protein present is then calculated from the nitrogen concentration of the food.

#### 2.6.2 Procedure

Two grams each of the samples were digested with sulfuric acid to decompose it and convert nitrogen to ammonium sulfate. The digestion was speeded up by adding Kjeldahl catalyst tablets to increase the boiling point. The solution was cooled and concentrated sodium hydroxide added to make the solution alkaline and distilled into a weak acid (boric acid) containing methyl red indicator until the solution turned from red to green. Following distillation, ammonia was trapped as ammonium borate and quantified by titrating with a strong standard hydrochloric acid (0.01 N) until solution turned from green to wine to measure the nitrogenous content. The amount of crude protein was calculated by multiplying the % nitrogen found by 6.25 (%) (CP = % Nitrogen × 6.25) (AOAC, 2003).

### 2.7 Crude fat or ether extract determination

#### 2.7.1 Principle

A dried ground sample is extracted with diethyl ether which dissolves fats, oils, pigments, and other fat soluble substances. The ether is then evaporated from the fat solution. The resulting residue is weighed and referred to as ether extract or crude fat.

#### 2.7.2 Procedure

Beaker was placed in an oven at 80°C for 10 min and then removed and placed in a desiccator to cool. Two grams dried food sample was weighed into the fat beaker, a glass thimble full of anhydrous diethyl either was added to the beaker and placed on the butt‐type extraction apparatus. This was then boiled at high temperature for approximately 4 hr to evaporate the ether, condense and allowed to pass through the sample, extracting ether soluble materials. The extract was collected in a beaker, allowed to cool and the porous thimble removed with contents saved for crude fiber determination. Ether was distilled and collected in another container until beaker was almost dry and the remaining ether extract was then dried in oven at 80°C for 3 min, cooled in the desiccator and weighed to measure the ether extract content (AOAC, 2003).

$$\text{Lipid}\;\left(\%\right)=\frac{\text{Weight of fat/oil}}{\text{Weight of sample}}\times 100$$

### 2.8 Crude fiber determination

#### 2.8.1 Principle

Two boiling processes simulate the pH conditions of the digestive tract, acidic in the stomach, and alkaline in the small intestine.

#### 2.8.2 Procedure

The residue obtained from ether extract was further treated with 1.25% sulfuric acid and 1.25% of sodium hydroxide under heating for 30 min. The content was ashed in a muffle furnace and reweighed (AOAC, 2003).

$$\text{Crude fiber}\;\left(\%\right)=\frac{\text{Weight of fibre}}{\text{Weight of sample}}\times 100$$

### 2.9 Determination of crude carbohydrates

Crude carbohydrates were determined according the method of AOAC (2003).

#### 2.9.1 Principle

Defatted samples of the test foods were hydrolyzed using three enzymes (heat stable α‐amylase, protease, and amyloglucosidase). These enzymes hydrolyze the carbohydrates and proteins in the test foods under incubation conditions with phosphate buffer.

#### 2.9.2 Procedure

Four milliliters of heat stable α‐amylase (0.5 g of α‐amylase in phosphate buffer) was added to one gram each of the defatted samples (*W*_1_) and allowed to incubate at 100°C for 30 min. Two milliliters of protease (pepsin) was added to the samples after incubation and the pH adjusted to 7.5 using 0.1 mol/L NaOH and incubated again at 60°C for 30 min. After which 2 ml of amyloglucosidase was added to the samples and the pH adjusted to 4.5. The samples were incubated at 60°C for 30 min and the carbohydrates in the samples precipitated using 80% ethanol and filtered using Whatman filter paper. The residues were washed repeatedly using 50% acetone and 50% ethanol, while the samples were dried at 50°C for 24 hr (*W*_2_).

#### 2.9.3 Calculations

$$\%\;\text{Carbohydrate}=\frac{W_{1}-W_{2}}{W_{1}+W_{3}}\times 100$$

$$W_{1}=\text{Initial weight of sample (1g)}$$

$$W_{2}=\text{Weight after hydrolysis}$$

$$W_{3}=\text{Lipid content in sample before lipid extraction}.$$

### 2.10 Determination of serving sizes

The GI test is based on 50 g in each test food of available carbohydrate, defined as:

Total carbohydrate−dietary fiber

Therefore, the portion size of each test food could vary according to the quantity of carbohydrate available in that food. Fifty grams of available carbohydrate was calculated from the results obtained from the proximate analysis of the test samples; the weight of the samples that would deliver fifty grams of available carbohydrates. The dry weight was determined using the calculation below.

Dry weight (DW)=100−moisture content

$$\begin{array}{c} \text{Weight of carbohydrate, in 100}\;\;\;\text{g dry weight}\\ =\frac{\%\;\text{CHO of test sample}}{100}\times \text{DW} \end{array}$$

### 2.11 Selection of subjects

Two hundred and forty (240) subjects (120 males and 120 females), aged between 18 and 30 years, were selected for the study and all subjects gave informed consent to participate. All subjects were recruited from Igbinedion University, Okada, Edo State, Nigeria, for voluntary participation in the study. The subjects chosen were healthy, with a body mass index of less than 25 kg/m²; they were also nonsmokers, not pregnant or diabetic and none of them had a family history of diabetes. The subjects were given full details of the study protocol and had opportunity to ask questions before the study. The protocol and procedures employed were reviewed and approved by the Ethics Committee of the College of Medical Sciences, University of Benin, Nigeria. The procedures followed were also in accordance with the ethical standards of the responsible committee on human experimentation and with the Helsinki Declaration of 1975, as revised in 2008.

The subjects were randomized into three groups (A, B, & C) of 80 individuals each (40 males and 40 females). The subjects were asked not to undertake vigorous activities on the day before the test and to avoid caffeine containing drinks 24 hr before the test; instructions concerning meals of the previous day were not provided, because the fat and carbohydrate content of the evening meal before GI testing does not influence the blood glucose response.

The foods consumed by each group are as described below;

All groups (A, B and C): Consumed the Standard Food (Glucose).

Group A Subjects: Consumed Test Food 1 (Rice and Beans)

Group B Subjects: Consumed Test Food 2 (Rice and Plantain)

Group C Subjects: Consumed Test Food 3 (Beans and Plantain).

### 2.12 Anthropometric measurements

All anthropometric measurements were taken after a 12‐hr fast with the subjects wearing light clothes and no shoes. Body Mass Index (BMI) was calculated as the weight in kilograms divided by the square of the height in meters (kg/m^2^).

#### 2.12.1 Weight measurement

To ensure reliable measurements of body weight using the mechanical bathroom scale (HANA mechanical bathroom scale; P.M.HANA, Central Hong Kong, Hong Kong) the scale was zeroed before the respondent stepped onto it. The respondents were asked to remove any “heavy” items from their pockets and any heavy items of clothing. They were asked to look straight ahead and stay still on the scales. The needle/digital screen was allowed to settle before the measurement was recorded. The body weight (kg) was measured to the nearest 0.5 kg (Ambrosini et al., 2009).

#### 2.12.2 Height measurement

Height measurement was taken using a “drop down” tape fixed at about 2 m on a wall. The respondents were asked to remove their shoes and stand with their back to the wall, looking directly forward. The back of their feet, calves, upper back and the back of their head were in contact with the wall. They were positioned directly underneath the drop down measuring tape. The measuring tape was lowered until it rested gently on the top of the respondent's head and the height (m) to the nearest 0.5 cm was recorded (Ambrosini et al., 2009).

### 2.13 Portion sizes

The GI test is based on 50 g in each test food of available carbohydrate. The portion size of each test food could vary according to the quantity of carbohydrate available in that food. The contributions of the component food in the test foods, making up the 50 g is as follows;

Test food 1/Group A (Rice and Beans)‐Rice30%and Beans20%

Test food 2/Group B (Rice and Plantain)‐Rice30%and Plantain20%

$$\begin{array}{cc} \text{Test food 3/Group C (Beans and Plantain)}-\; & \text{Beans}\;25\;\%\;\\ & \text{and Plantain}\;25\%. \end{array}$$

### 2.14 Determination of glycemic index

GI was determined by the method of Wolever et al. (2001). The GI of the test foods were determined by feeding each test food to 80 healthy individuals. Blood was obtained by finger prick using the Accu‐Chek Softclix lancing device (Accu‐Check^R^ Active). The finger was chosen at random. Before the finger prick, the subjects were encouraged to warm their hands (by rubbing the palms together) to increase blood flow. Blood samples were collected at intervals of 30 min for 2 hr (0, 30, 60, 90 and 120 min). The blood glucose concentrations were determined using a glucometer with a glucose test strip (Accu‐chek Active).

#### 2.14.1 Principle

Analysis was based on glucose oxidase method.

#### 2.14.2 Procedure

A sample of fresh blood was placed on the test pad containing an enzyme system having glucose oxidase and peroxide activity. The enzyme system reacted with glucose and released hydrogen peroxide. The pad on the test strip also contained an indicator which reacted with hydrogen peroxide in the presence of peroxidase to form a color proportional in intensity to the glucose concentrations in the samples.

##### Day 1

The study started in the morning after an overnight fast by the individuals. A fasting blood sample was taken at 0 min; then immediately after this, the subjects consumed 50 g standard food (50 g of glucose powder dissolved in water) in a comfortable place. The standard food was taken with 200 ml of water. More blood samples were taken at 30, 60, 90, and 120 min. The blood glucose concentrations were determined immediately using the glucometer.

##### Day 2

After an overnight fast, the test foods were consumed by each group (of 80 subjects). Blood samples were then taken at 0, 30, 60, 90, and 120 min. The blood glucose concentrations were determined immediately using the glucometer.

### 2.15 Calculation of glycemic index

The incremental area under the blood glucose response curve (IAUC), ignoring the area beneath the baseline was calculated geometrically. The IAUC calculated for each test meal eaten by each subject was expressed as a percentage of the mean IAUC for the standard food eaten by the same subject as follows:

$$\text{GI}=\frac{\text{Incremental blood glucose area of test food}}{\text{Incremental blood glucose area of reference food}}\times 100$$

### 2.16 Calculation of glycemic load

The glycemic load, which assesses the total glycemic effect of the diet and has proved very useful in epidemiologic studies, is the product of the dietary GI and total dietary carbohydrate;

$$\text{GL}=\frac{\text{GI}}{100}\times \text{Carbohydrate content (g)}.$$

### 2.17 Statistical analysis

Statistical analysis was performed using Graphpad Prism 7.0. The paired student *t*test was used to compare the mean of the IAUC of the standard food with each of the test foods. Statistical significance was set at *p* < .05.

## 3 RESULTS

### 3.1 Proximate composition of the test foods

The first stage involved in the calculation of GI values of the test foods was the determination of the proximate composition of the selected foods.

The preparation of test food 3 involved frying and as such had high‐fat content. The fiber analysis shows that beans had the lowest value (0.19%) while plantain had the highest (0.357%). Table 1 also shows that the % carbohydrate composition is highest in rice and lowest in beans, while the % protein composition is highest in beans and lowest in plantain.

**Table 1:** Proximate composition of the test foods (100 g)

	Rice	Beans	Plantain
Moisture (%)	16.14 ± 0.079	18.73 ± 0.095	16.99 ± 0.089
Protein (%)	0.13 ± 0.012	69.49 ± 0.113	0.097 ± 0.018
Ash (%)	0.34 ± 0.012	0.147 ± 0.019	0.507 ± 0.015
Fiber (%)	0.24 ± 0.015	0.19 ± 0.012	0.357 ± 0.012
Lipids (%)	0.107 ± 0.009	0.09 ± 0.017	10.323 ± 0.217
Carbohydrate (%)	63.71 ± 0.074	10.50 ± 0.003	32.98 ± 0.265

Values represent mean ± SEM (*n* = 3).

### 3.2 Portion sizes

The portion sizes of the test foods are 126.75 g for test food 1 (rice and beans), 153.5 g for test food 2 (rice and plantain), and 65.8 g for test food 3 (beans and plantain).

### 3.3 Physical characteristics of the study population

The subjects for this study were selected according to the specific criteria stated in the Table 2.

**Table 2:** Physical characteristics of the study population

Characteristics	Mean ± SEM
Age (years)	21.0 ± 0.547
Height (m)	1.66 ± 1.784
Weight (kg)	60.4 ± 2.021
BMI (kg/m^2^)	21.5 ± 0.498
WC (cm)	72.5 ± 1.761

BMI, body mass index; WC, waist circumference.

Values represent mean ± SEM (*n* = 240).

The physical characteristics of the study subjects were as follows; mean weight 60.4 ± 2.021 kg, BMI 21.5 ± 0.498 kg/m^2^ , and WC 72.5 ± 1.761 cm (Table 2).

For the standard food, there was a steady fall in glucose level after 30 min while the glucose level for test food 1 increased steadily (Table 3).

**Table 3:** Blood glucose concentrations (mg/dl) at time intervals (0, 30, 60, 90, 120 min) for group A (test food 1)

	Glucose	Test food 1
0 min	72.625 ± 4.009	77.875 ± 3.125
30 min	125.275 ± 14.481	87.25 ± 2.596
60 min	107.375 ± 12.365	91.00 ± 3.268
90 min	94.875 ± 6.993	91.125 ± 3.791
120 min	89.50 ± 5.134	91.25 ± 4.233

Values represent mean ± SEM (*n* = 80).

There were steady decreases in blood glucose concentrations for the standard food (glucose) and test food (Table 4).

**Table 4:** Blood glucose concentrations (mg/dl) at time intervals (0, 30, 60, 90, 120 min) for group B (test food 2)

Time (min)	Glucose	Test food 2
0 min	72.875 ± 3.796	76.375 ± 2.017
30 min	122.875 ± 2.985	110.25 ± 7.228
60 min	106.125 ± 3.912	95.375 ± 4.555
90 min	106.25 ± 12.209	90.625 ± 5.916
120 min	96.125 ± 7.657	84.50 ± 2.952

Values represent mean ± SEM (*n* = 80).

Table 5 shows initial rise in blood glucose levels in test food 3, which later decreases.

**Table 5:** Blood glucose concentrations (mg/dl) at time intervals (0, 30, 60, 90, 120 min) for group C (test food 3)

Time (min)	Glucose	Test food 3
0 min	69.875 ± 1.445	83.75 ± 2.469
30 min	125.375 ± 6.456	95.75 ± 3.604
60 min	104.0 ± 7.737	97.125 ± 2.401
90 min	111.0 ± 18.811	93.625 ± 3.354
120 min	98.75 ± 9.013	81.625 ± 2.809

Values represent mean ± SEM (*n* = 80).

### 3.4 GI, GL, and classification of test foods

Table 6 shows the GI and classification of the three test foods. These results showed that the GI values ranged from 89.74 to 86.60, which classified them all as high‐GI foods. Test food 1 had the lowest GI value (86.60) while test food 2 had the highest value (89.74). The GL value ranged from 43.30 to 44.87 putting all the test foods in the high‐GL category.

**Table 6:** GI classification of the three test foods

Test foods	Available CHO (g)	Portion size (g)	GI value	GL value	Classification
Test food 1 (rice and beans)	50	126.75	86.60	43.30	High
Test food 2 (rice and plantain)	50	153.5	89.74	44.87	High
Test food 3 (beans and plantain)	50	65.8	86.93	43.47	High

### 3.5 Incremental area under the curve for the standard and test foods

Table 7 shows the IAUC for the three test foods. Significant differences were found in the IAUC between the standard and the test food for each group.

**Table 7:** Incremental area under the blood glucose response curve (IAUC) for test foods

Group	Test food	IAUC ± SEM	*p* value
Group A	Glucose	122.61 ± 12.48	
	Test food 1 (Rice and beans)	106.18 ± 4.05	.2631
Group B	Glucose	125.93 ± 8.69	
	Test food 2 (Rice and plantain)	113.01 ± 6.41	.0475
Group C	Glucose	127.41 ± 13.38	
	Test food 3 (Beans and plantain)	110.76 ± 3.65	.1916

Values represent mean ± SEM (*n* = 80).

### 3.6 Glycemic response in food

**Figure 1:**
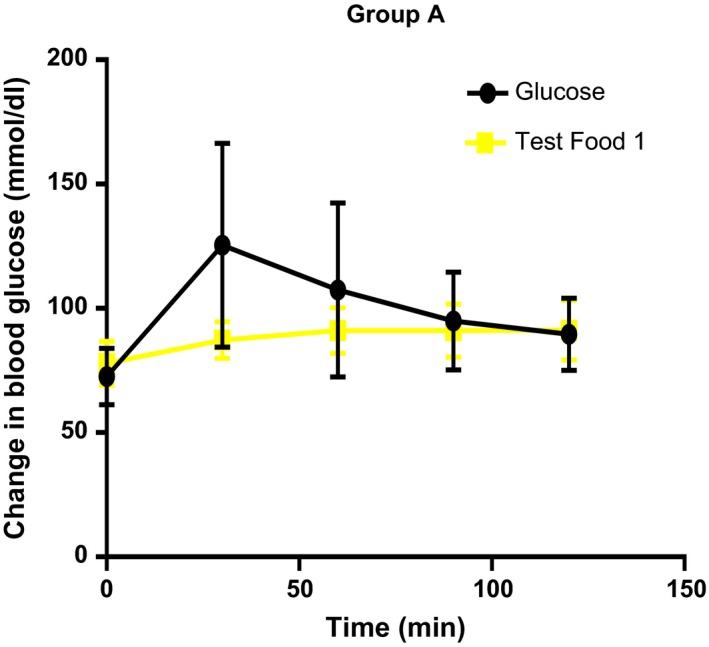
The incremental increase in blood glucose at 30 min was significantly different between test food 1 (rice and beans) and the standard food (*p* = .0360 mean *= *95.75 mg/dl)

**Figure 2:**
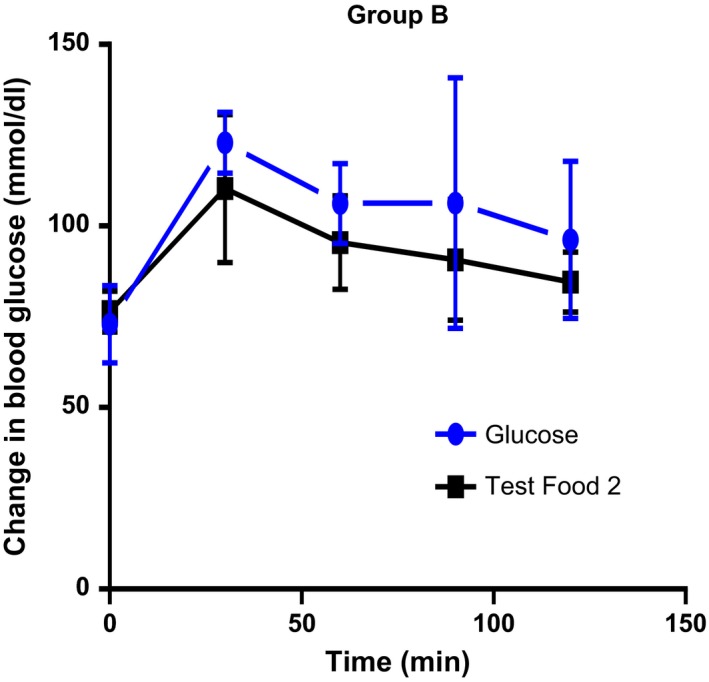
There was no significant difference in the incremental blood glucose between the standard and test food

**Figure 3:**
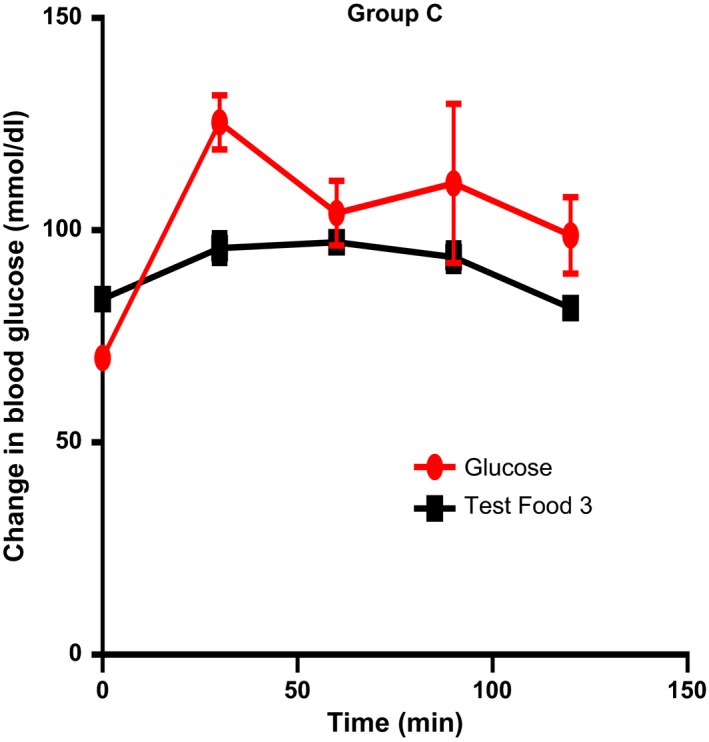
At, 30 min, test food 3 showed a significant difference in incremental blood glucose level (*p* = .0068, mean = 87.25 mg/dl) (Figure 3)

The mean incremental areas under the glycemic response curves for the standard and test foods are shown below (Figures 1, 2, 3). The differences in glucose response between the test foods were analyzed using *t* test.

## 4 DISCUSSION

The incorporation of low‐glycemic index carbohydrate foods such as legumes in the diet has been shown to reduce the post‐prandial and 24 hr glucose response in individuals with diabetes (Simpson, Simpson, & Lousley, 1981). Glycemic responses to meals may vary significantly with the method of cooking, heat utilized, amount of water and the time of cooking (Collings, Williams, & MacDonald, 1981; Vaaler, Hanssen, & Aagenaes, 1984). GI is not only the measure of the carbohydrate absorption in the small intestine directly, but also indicates the effect of other factors in the foods tested that can influence the rate of carbohydrate absorption in the small intestine. This study shows that there are significant differences in the glycemic responses to different mixed meals.

A mixed meal of carbohydrate, protein and fat will have a different and variable glucose response depending on the proportion of each nutrient. In this study, the constituent of each test food was served in different proportions; test food 1 and 2 had more rice than beans or plantain as is normally eaten in the locale. The greater proportion of rice, a high‐carbohydrate food, in both meals is likely responsible for the increase in the GI values.

Protein‐rich foods are known to increase insulin secretion without augmenting glucose concentrations (Krezowski, Nuttall, Gannon, & Bartosh, 1986; Nuttall, Mooradian, Gannon, & Bartosh, 1984; Simpson, McDonald, Wahlqvist, Altey, & Outch, 1985). Therefore, as more protein is taken in conjunction with carbohydrate, the insulin response will increase, whereas postprandial glucose will not change much. This could account for the reduced GI value seen in test food 3, which has a greater amount of protein (beans has a protein content of 69.49%). Also the plantain which was fried, could have contributed a high‐fat content to test food 3 which also decreases the GI value. Adding fat to a carbohydrate meal also enhances insulin secretion even though the plasma glucose response actually decreases. Comparing test food 1, which has a GI value of 86.60 and test food 2, which has a GI value of 89.74, with test food 1 having a lesser GI value, this could be attributed to the protein content in the mixture. Our findings show that the selected test foods (mixed meals) consumed in Okada Town have high‐GI values. The high‐GI food (mixed meal) consumed by the people may increase their risk of cardiovascular diseases. However, this may not be a common phenomenon since they often eat varieties of food some of which help in protecting against cardiovascular diseases. It is pertinent to know that the people have high dietary diversity score and the interactions between the various nutrients may bring about long‐term positive effects.

Different nutritional and physiological factors might have an effect on the blood glycemic response and the GI value of the mixed meals. Included among these factors is the digestibility rate of the starch, the interactions of starch absorption with the amount of fiber, fat and protein present, the proportion of the constituent nutrient and the cooking methods.

## 5 CONCLUSION

Our findings show that the selected test foods (mixed meals) consumed in Okada Town, Edo State, Nigeria, have high‐GI values.

## CONFLICT OF INTEREST

We the authors wish to confirm that there are no known conflicts of interest associated with this publication and there has been no significant financial support for this work that could have influenced its outcome.
